# Preparative immunotherapy with anti-OX40 and anti-CTLA4 improves the response to chemotherapy

**DOI:** 10.1186/2051-1426-2-S3-P207

**Published:** 2014-11-06

**Authors:** David J Friedman, Kristina Young, Jason R Baird, Benjamin Cottam, Talicia Savage, Pippa Newell, Melissa Kasiewicz, William Redmond, Brendan Curti, Todd Crocenzi, Michael J Gough, Marka Crittenden

**Affiliations:** 1 Earle A. Chiles Research Institute, Robert W. Franz Cancer Center, Providence Portland Medical Center, Portland, OR, USA; 2 Department of Radiation Medicine, Oregon Health & Science University, Portland, OR, USA; 3 The Oregon Clinic, Portland, OR, USA

## 

Recent studies have reported that decreased T cell infiltrate alone, or co-ordinate with increased macrophage infiltrate, correlate with decreased survival in a range of cancers, including patients with pancreatic cancer. Importantly, in mouse models of pancreatic cancer, therapies that decrease tumor-associated macrophage infiltrates improve the response to chemotherapy, matching data in mammary cancer models. We have previously demonstrated that immunotherapy with agonistic antibodies to OX40 was able to remodel tumors, resulting in increased CD8 infiltrate and as a consequence, decreased macrophage infiltrate. Similarly, others have shown that blocking antibodies to CTLA-4 result in increased T cell infiltrate to tumors, both in mouse models and in patients. We hypothesize that immunotherapies that remodel the tumor immune environment will increase the response to chemotherapy.

To test our hypothesis, we used the Panc02 mouse model of pancreatic adenocarcinoma that forms a highly chemo- and radio-resistant tumor in immunocompetent mice, with extensive stromal involvement and diminished drug penetrance compared to more immunogenic tumors. We demonstrate that systemic immunotherapy with anti-OX40 and anti-CTLA4 transiently changed the polarization of macrophages in tumors as determined by decreased arginase expression, and delivery of gemcitabine chemotherapy during the window of changed macrophage polarization resulted in significantly improved tumor control and survival. However, we also demonstrate that combination immunotherapy resulted in Th2 differentiation in tumor-bearing mice, associated with IL-4 production by activated CD4 T cells, potentially driving the strong rebound of arginase expression in tumor macrophages at later time points. Inhibiting IL-4 in vivo improved the efficacy of immunochemotherapy by significantly extending survival. These data demonstrate that preparative immunotherapy is a novel treatment option to improve the efficacy of chemotherapy where the immune environment is poor, and may increase response rates in cancers with negative immunology.

